# Vacuolar ATPase ‘a2’ isoform exhibits distinct cell surface accumulation and modulates matrix metalloproteinase activity in ovarian cancer

**DOI:** 10.18632/oncotarget.2902

**Published:** 2015-01-21

**Authors:** Arpita Kulshrestha, Gajendra K. Katara, Safaa Ibrahim, Sahithi Pamarthy, Mukesh K. Jaiswal, Alice Gilman Sachs, Kenneth D. Beaman

**Affiliations:** ^1^ Department of Microbiology and Immunology, Rosalind Franklin University of Medicine and Science, North Chicago, IL, USA; ^2^ Department of Microbiology and Immunology, Faculty of Pharmacy, Cairo University, Egypt

**Keywords:** Vacuolar ATPase, a2 isoform, ovarian cancer, invasion, cortactin, MMP

## Abstract

Tumor associated vacuolar H+-ATPases (V-ATPases) are multi-subunit proton pumps that acidify tumor microenvironment, thereby promoting tumor invasion. Subunit ‘a’ of its V0 domain is the major pH sensing unit that additionally controls sub-cellular targeting of V-ATPase and exists in four different isoforms. Our study reports an elevated expression of the V-ATPase-V0a2 isoform in ovarian cancer(OVCA) tissues and cell lines(A2780, SKOV-3 and TOV-112D). Among all V0’a’ isoforms, V0a2 exhibited abundant expression on OVCA cell surface while normal ovarian epithelia did not. Sub-cellular distribution of V-ATPase-V0a2 confirmed its localization on plasma-membrane, where it was also co-associated with cortactin, an F-actin stabilizing protein at leading edges of cancer cells. Additionally, V0a2 was also localized in early and late endosomal compartments that are sites for modulations of several signaling pathways in cancer. Targeted inhibition of V-ATPase-V0a2 suppressed matrix metalloproteinase activity(MMP-9 & MMP-2) in OVCA cells. In conclusion, V-ATPase-V0a2 isoform is abundantly expressed on ovarian tumor cell surface in association with invasion assembly related proteins and plays critical role in tumor invasion by modulating the activity of matrix-degrading proteases. This study highlights for the first time, the importance of V-ATPase-V0a2 isoform as a distinct biomarker and possible therapeutic target for treatment of ovarian carcinoma.

## INTRODUCTION

An acidic microenvironment is known to be crucial for tumor growth, progression and chemo-resistance [1–2]. Vacuolar H+-ATPases (V-ATPase) are the key proton pumps responsible for both the establishment and the maintenance of the acidic pH of tumors [3]. Under normal physiological conditions, these ion exchangers contribute to maintaining pH homeostasis, regulation of organelle pH [4–6] and inducing extracellular acidosis in specialized tissues [7–8]. An abundance of V-ATPase expression has been found on tumor cell surface that critically influences the malignant behavior of cancer by activating the matrix degrading proteases [9–12]. Sub-cellular V-ATPases also contribute to vesicular trafficking of these proteases to the cell surface as well as their activation. Additionally, V-ATPases also regulate other signalling molecules such as Wnt, Notch etc [13]. New strategies are therefore being explored for selective inhibition of V-ATPases to control tumor growth and invasion [3, 14].

The mammalian V-ATPase molecule contains 13 different subunits organized into an ATP-hydrolytic domain (V1) and a membrane bound proton-translocation domain (V0) that work together as a rotary machine [15]. The 116-kDa V0 ‘a’ is the largest subunit of the V0 domain and plays an important role in proton transport. V0‘a’ subunit is of special interest as it also contains information to target V-ATPases to different sub-cellular membranes [16–17]. It consists of four isoforms, namely V0a1, V0a2, V0a3, and V0a4 that are encoded by different genes with tissue-specific expression. In normal physiological cells, the V0a1 isoform is expressed on synaptic and clathrin-coated vesicles in brain [18], whereas V0a2 is abundantly expressed in kidney, lung, spleen and reproductive organs [19–20]. The V0a3 isoform is strongly expressed on the plasma membrane of the mature osteoclasts [21]. The subunit a4 isoform is highly expressed in kidney cells for renal acid/base homeostasis [8].

In tumor cells, V0a1 and a2 expression has been found abundantly in breast cancer and was required for Rab27B dependent invasive growth [22]. Breast tumor cells employ the ‘V0a3’ subunit isoform to target V-ATPases to the plasma membrane, where they assist in tumor cell invasion [17]. A number of studies have demonstrated that cancer cells are sensitive to V-ATPase inhibitors [14], and recently it was suggested that cancer growth and metastasis could be blocked in mice by knocking down the a3-subunit in B16 melanoma cells [23]. With regard to V0a2, our previous studies have shown that the V0a2 isoform is highly expressed on breast cancer and melanoma cell surfaces and its cleaved ‘N’ terminal domain has a role in cancer-related inflammation [24–25]. The V0a2 isoform expression therefore is known to have cancer-promoting effects.

Ovarian cancer (OVCA) is the most lethal gynecological neoplasia. It globally accounts for over 100,000 female deaths out of 2,04,000 diagnosed patients per year [26–27], which is attributable to delayed detection and chemo-resistance [28]. Therefore, exploration of new cancer associated molecules as early biomarkers and possible therapeutic targets is crucial for development of effective strategy for controlling the disease. The expression and involvement of different V-ATPase ‘a’ subunit isoforms in ovarian cancer still remains largely unknown. Moreover, the differences in isoform expression patterns are not completely understood. We report, for the first time, the expression pattern of the V-ATPase ‘a’ subunit isoforms in OVCA. Our results demonstrate that the V-ATPase-V0a2 isoform is distinctly up-regulated on the ovarian tumor cell surface among all the ‘a’ subunit isoforms, both in the tissues as well as OVCA cell lines. Additionally, V0a2 localizes with the known components of the cellular invasive machinery on plasma membrane of OVCA cells and also with endosomal compartments. Further, the targeted inhibition of V0a2 using monoclonal anti-V0a2 antibody decreased the matrix metalloproteinase (MMP-2 and MMP-9) activity in OVCA cells. The study highlights the importance of V-ATPase V0a2 subunit as a distinct biomarker and possible therapeutic target for ovarian carcinoma.

## RESULTS

### V-ATPase-V0a2 isoform is over-expressed in ovarian cancer tissues

The V-ATPase ‘a’ subunit isoform specific antibodies were generated and characterized in our lab as described in materials and methods section. These antibodies were generated against the unique regions of human V-ATPase-V0a1, V0a2 or V0a3 and did not cross react with each other [supplementary Figure S1(A–D)].

**Figure 1:**
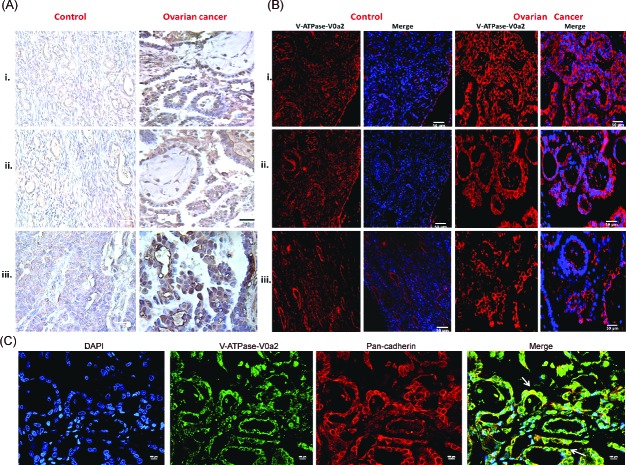
Ovarian cancer tissues express high levels of V-ATPase-V0a2 isoform Anti-V0a2 monoclonal antibody directed against 488–510 amino acids of trans-membrane region (2C1) was employed to study V0a2 expression. **(A)** Immuno-histochemical staining of V-ATPase-V0a2 isoform in **Control ovarian tissues-** (i, ii) Normal ovary and (iii) benign ovarian tumor; **Ovarian cancer** panel shows V0a2 staining in (i)- grade I serous cystedenocarcinoma. (ii, iii) grade II serous cystedenocarcinoma. The ovarian cancer tissues have increased number of V-ATPase V0a2-positive cells (brown) compared to normal ovarian tissue. Original magnification- × 400; scale bars- 50 μm **(B)** Immunofluorescence analysis in control and ovarian carcinoma tissues also revealed higher V0a2 expression (in red) compared to control. Nuclear staining with DAPI. Original magnification- × 400; scale bars- 50 μm **(C)** Immunofluorescence analysis of V-ATPase-V0a2 (in green) and pan cadherin (in red) for plasma membrane staining in serous cystedenocarcinoma tissue. Merge areas shown by arrow (in yellow). Nuclear staining with DAPI. Original magnification: × 800; scale bars, 100 μm. Representative images from three independent experiments are shown. The following Tissue Array was used: human ovary tumor tissue array; Biochain (Cat no: Z7020088).

To characterize V-ATPase-V0a2 expression in ovarian tumors, we used the anti-V0a2 antibody [24–25] that recognizes specific epitopes of V0a2 isoform. Human ovarian carcinoma tumor tissue arrays were employed for these studies. The ovarian cancer tissues showed increased number of V-ATPase V0a2-positive cells (brown) compared to normal ovarian tissue [Figure 1(A)]. V0a2 expression was evident on the plasma membrane as well as the cytoplasm of metastatic tissues. These results are in line with our previous studies where V0a2 was shown to be highly expressed on the tumor cell surface in breast cancer and melanoma tissues [25]. Immunofluorescence analysis also revealed abundant expression of V-ATPase-V0a2 in OVCA tissues [Figure 1(B)]. Further, the immunofluorescence analysis showed co-association of V0a2 with the cell surface marker, pan cadherin [Figure 1(C)]. Interestingly, V0a2 exhibited very low expression in benign tumor tissues and was expressed distinctly on metastatic tumors [Figure 1(A) control panel (iii)] indicating its role in tumor metastasis and its utility as a biomarker for metastatic OVCA tumors.

### V-ATPase-V0a2 expression is highly elevated on ovarian cancer cell surface

**Figure 2:**
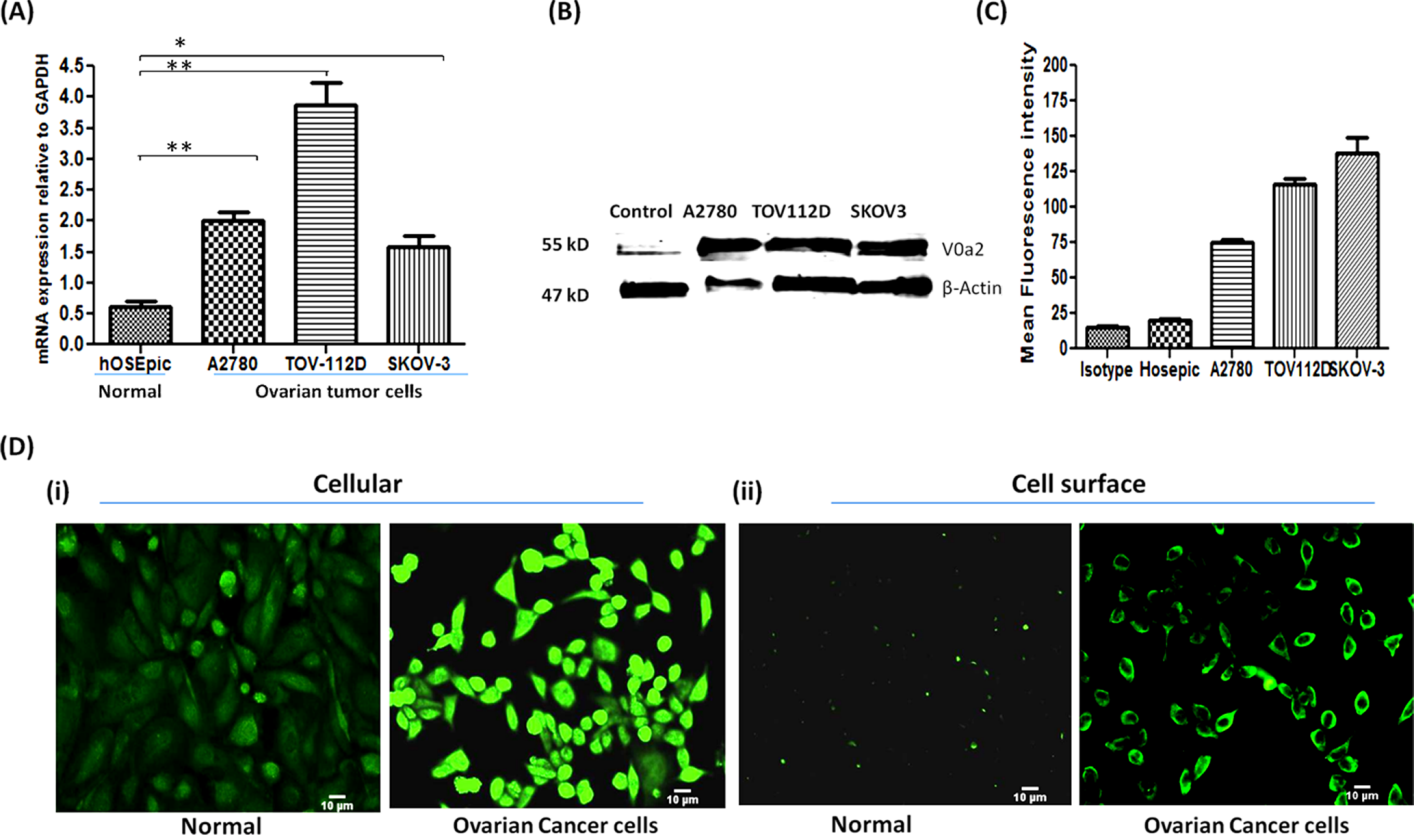
Ovarian carcinoma cell lines exhibit high expression of V-ATPase-V0a2 on cell surface **(A)** Real time PCR analysis revealed higher relative mRNA levels of V-ATPase-V0a2 isoform in ovarian cancer cell lines compared to normal ovary epithelia. The Ct values were normalized against the Ct values for GAPDH from the same preparation. The data are provided as mean ± SD from 3 independent experiments. (***p < 0.0001, **p < 0.001, *p < 0.05). **(B)** Total protein from ovarian tumor cell lines and respective normal ovarian epithelial cells were immunoblotted with anti-V0a2 (2C1) that indicated higher expression levels of V0a2. **(C)** Surface V0a2 expression on human ovarian cancer cell lines (TOV-112D, A2780, SKOV-3) relative to normal human ovarian epithelial cells (hOSEpic) cells was examined by flow cytometry. Histogram showing geometric mean fluorescence intensities of anti-V0a2-stained cells divided by isotype ± s.e.m (*n* = 6). All experiments were repeated at least twice in duplicate. **(D)** For Immunofluorescence analysis, 0.5 × 10^4^ TOV-112D cells were cultured in chamber slides, fixed with paraformaldehyde and incubated with anti-V0a2 antibody (green) and examined microscopically. Image (i) shows cellular V0a2 and (ii) surface V0a2 expression in TOV-112D ovarian cancer cell line compared to normal ovary epithelia. Original maginification-× 200; Scale bars-10 μm. Representative images from four independent experiments performed in duplicate are shown.

In order to decipher the V0a2 expression levels in ovarian cancer cells, we employed three OVCA cell lines (A2780, SKOV-3 and TOV-112D). mRNA expression profiling by real time RT-PCR analysis revealed that in comparison to normal ovarian epithelial cells (hOSEpic), V0a2 mRNA was significantly elevated (upto 4 fold; *p* < 0.001) in all three observed OVCA cell lines [Figure 2(A)].

Our previous studies have shown that the VATPase-V0a2 isoform [earlier known as Regeneration and Tolerance Factor (RTF) or TJ6] is a 70kDa surface protein expressed in broad spectrum of tissues. Moreover, RTF is cleaved to yield a membrane-bound 50-kDa protein and a secreted, biologically active 20-kDa fragment (soluble RTF) [29–31; 24]. At the protein level, the western blot analysis revealed high levels of total V0a2 protein (depicted by 50 kDa band) in OVCA cell lines compared to normal ovarian cells [Figure 2(B)]. To further validate this, the protein expression profile of cellular as well as surface V0a2 was assessed by flow cytometry analysis. The three observed OVCA cells abundantly expressed V0a2 isoform compared to normal epithelial cells of the ovary [data not shown]. To assess the surface expression of the V0a2 isoform, we stained the non-permeabilized OVCA cells. Interestingly, V0a2 was selectively expressed on the OVCA cell surface while the normal ovarian epithelia showed very low if any expression on the surface [Figure 2(C)]. Immunofluorescence analysis also showed high cellular V0a2 expression in the OVCA cells compared to normal ovarian epithelial cells [Figure 2(Di)] Further, a prominent V0a2 surface expression was seen distinctly on OVCA cells and no expression was observed on normal ovarian epithelial cells [Figure 2 (Dii)] in non-permeabilized cells.

### Ovarian cancer cells exhibit V-ATPase-V0a2 plasma membrane localization and association with components of cellular invasion

**Figure 3:**
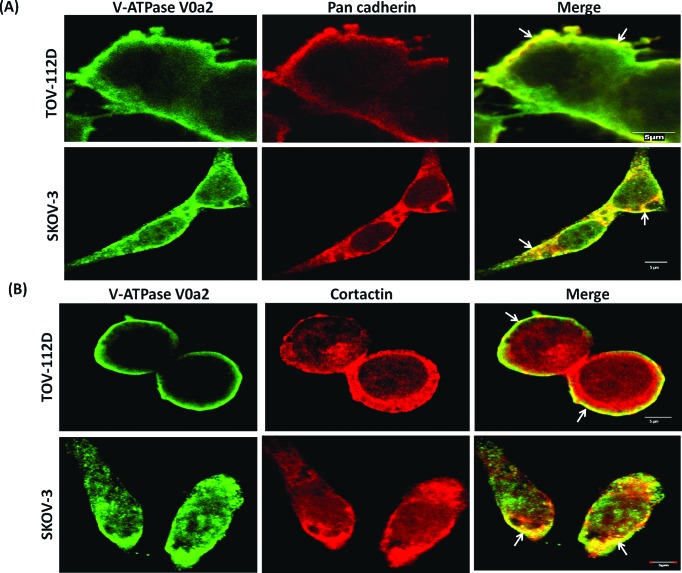
V-ATPase-V0a2 isoform exhibits association with invasion associated proteins on ovarian cancer cell surface Confocal imaging of **(A)** V-ATPase-V0a2 (in green) and pan cadherin (in red) for plasma membrane staining in TOV-112D and SKOV-3 ovarian carcinoma cells. Merged images (yellow regions, shown with arrow) show the co-localization of V-ATPase-V0a2 with pan cadherin. **(B)** Representative image showing expression of V-ATPase-V0a2 (in green) and cortactin (in red) in TOV-112D and SKOV-3 ovarian carcinoma cells. Merged images (yellow regions, shown with arrow) indicate the co-association of V0a2 in some regions of the plasma membrane with cortactin, a component of the cellular invasion apparatus at the leading edge. Original maginification-× 600; scale bars; 5 μm. No V0a2 co-association with cortactin could be observed intracellularly. Images were acquired using the Fluoview FV10i confocal laser-scanning microscope (Olympus). Olympus Fluoview software version 4.1 (Olympus) was used to capture and analyze the images. Representative images from four independent experiments are shown.

We further determined the sub-cellular localization of the V-ATPase-V0a2 in OVCA cell lines by confocal microscopy. The V0a2 is suggested to be predominantly located on the plasma membrane in ovarian cancer cells since it exhibited co-localization with pan-cadherin [Figure 3(A)]. This indicates the association of this specific isoform with surface V-ATPases that provide acidic extracellular environment promoting tumor metastasis.

An important characteristics of ovarian cancer is invasion and metastasis. Actin assembly plays a pivotal role in cell migration and invasion [32]. We found that V-ATPase V0a2 localized with F-actin rich structures as stained with phalloidin [Supplementary figure S2]. The branched actin regulator, cortactin, is a Src kinase substrate and Arp2/3 binding protein that stabilizes the actin filaments at the leading edge of tumor cells. The leading edges or the invadopodia on the cancer cell plasma membranes are the domains for protease release that are required for matrix degradation and cell invasion [33]. We therefore investigated whether the V0a2 isoform is co-associated with cortactin in OVCA cells. Analogous to a previous study [28] that reports V-ATPase and cortactin association, our data also suggests that in certain regions of the plasma membrane, V0a2 is associated with cortactin [Figure 3(B)].

**Figure 4:**
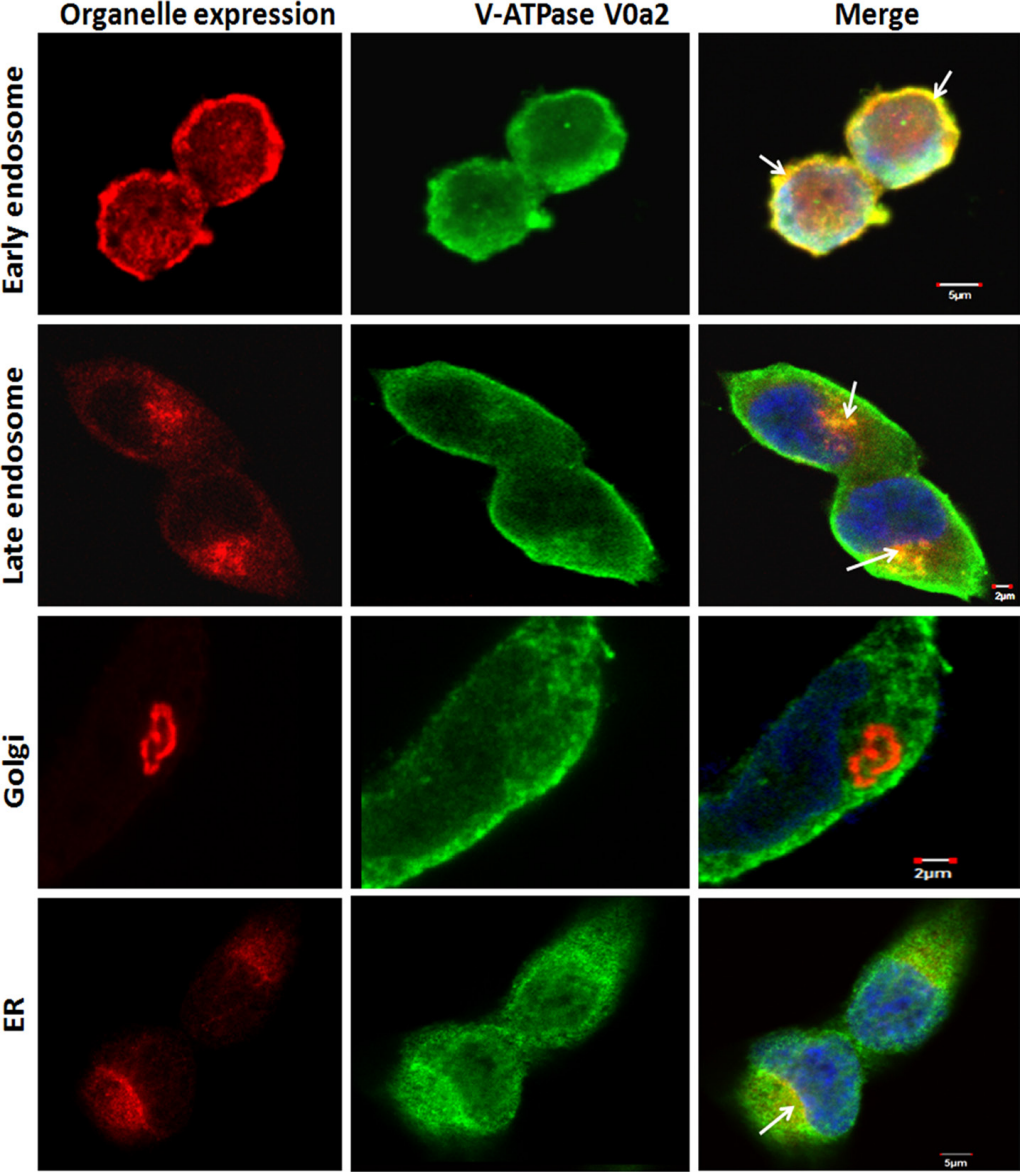
Sub-cellular localization of V-ATPase-V0a2 shows endosomal association in ovarian cancer cells Confocal microscopy analysis showing subcellular localization of vacuolar ATPase-V0a2 in ovarian cancer cell line, TOV-112D. Anti-Rab5 antibody stains the early endosomes (in red) while V0a2 is stained in green. Merged image indicates co-localization of V-ATPase-V0a2 with early endosomal machinery, as indicated by yellow areas shown with arrow in the merged image. Original magnification- × 600 bar represents 5 μm. Anti-Rab7 antibody stained the late endosome/lysosome (in red), while V0a2 isoform is stained in green. V0a2 exhibits co-expression with late endosomes as depicted by yellow regions (arrow) in merged image, original magnification- × 600; bar represents 2 μm. The V0a2 (in green) did not exhibit any co-association with Golgi using anti-Golph4 antibody (in red) original magnification- × 600; scale bar- 2 μm. Partial V0a2 (in green) association could be observed with ER marker, calnexin (in red), shown by arrow in merged image. The cells were co-stained with DAPI (blue). Images were acquired using the Fluoview FV10i confocal laser-scanning microscope. Olympus Fluoview software version 4.1 (Olympus) was used to capture and analyze the images.

The presence of this isoform was also evident in the early and late endosomal compartments [Figure 4]. However, no co-localization was observed with other endocytic compartments such as golgi and only partial association was observed with the endoplasmic reticulum [Figure 4].

### The expression pattern of V-ATPase V0a1 and V0a3 isoforms in OVCA cells

**Figure 5:**
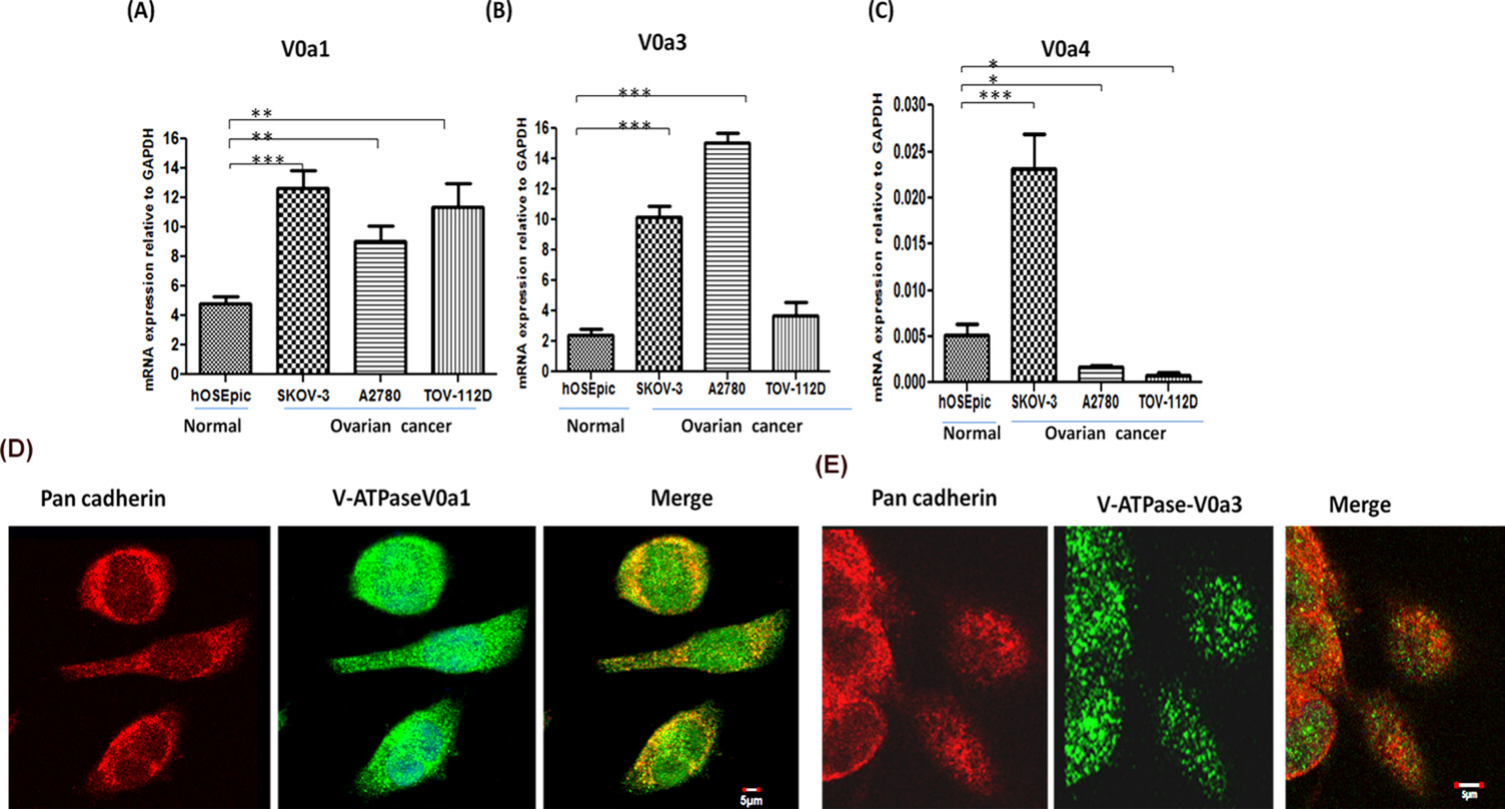
Expression profiling of V-ATPase ‘a’ subunit isoforms in ovarian cancer Real time PCR was used to quantify the relative mRNA amounts of V-ATPase V0‘a’ isoforms (V0a1/V0a3/V0a4) in ovarian cancer cell lines. The ovarian cancer cells exhibited higher mRNA levels of **(A)** V0a1 and **(B)** V0a3 isoforms in ovarian cancer cell lines compared to normal control epithelial cells of ovary. **(C)** V0a4 isoform levels were very low and variable in different cell lines. The Ct values were normalized against the Ct values obtained for GAPDH from the same preparation. The data are provided as Mean ± SD from 3 independent experiments. (***p < 0.0001, **p < 0.001, *p < 0.05). **(D)** V0a1 (green) exhibited some co-association with plasma membrane marker, pan-cadherin (red), merged image shows yellow area, scale bar 5 μm. **(E)** V0a3 (green) did not exhibit co-association with plasma membrane marker, pan-cadherin. Original magnification- X 600, scale bar 5 μm.

To further decipher the expression profile of other V-ATPase V0‘a’ isoforms (V0a1, a3, a4) in OVCA cells, real time RT-PCR was performed as described previously, using isoform specific primers. The expression of the V0a1 isoform was significantly elevated (*p* < 0.001) in all OVCA cells [Figure 5(A)] with up to 2.5 fold increase in V0a1 mRNA compared to normal epithelia of the ovary. Similarly, the expression of V0a3 was found significantly increased in A2780 and SKOV3 cell lines but this increase was not seen significant in the TOV112D cell line [Figure 5(B)]. The mRNA increase of V0a3 was the highest among all V0a isoforms (up to 15 fold) in OVCA cells with respect to normal cells. The mRNA levels of the V0a4 isoform was found to be significantly lower in 2 of the 3 reported cell lines (fold change ~ 0.125) compared to normal ovarian epithelia which, indicated that the V0a4 expression varies dramatically in different OVCA cells [Figure 5(C)]. V0a4 isoform was therefore excluded from further analysis. The protein expression profile of V-ATPase ‘a1/a3’ isoforms was assessed by western blot analysis [Supplementary figure S1(C)]. The confocal microscopy analysis revealed that V0a1 is co-associated with plasma membrane marker, pan-cadherin [Figure 5(D)]. The expression of V0a3 isoform was intracellular and did not show co-association with plasma membrane marker pan cadherin [Figure 5(E)]. Some association was observed with early endosomes using EEA-1 antibody [Supplementary figure S3].

**Figure 6:**
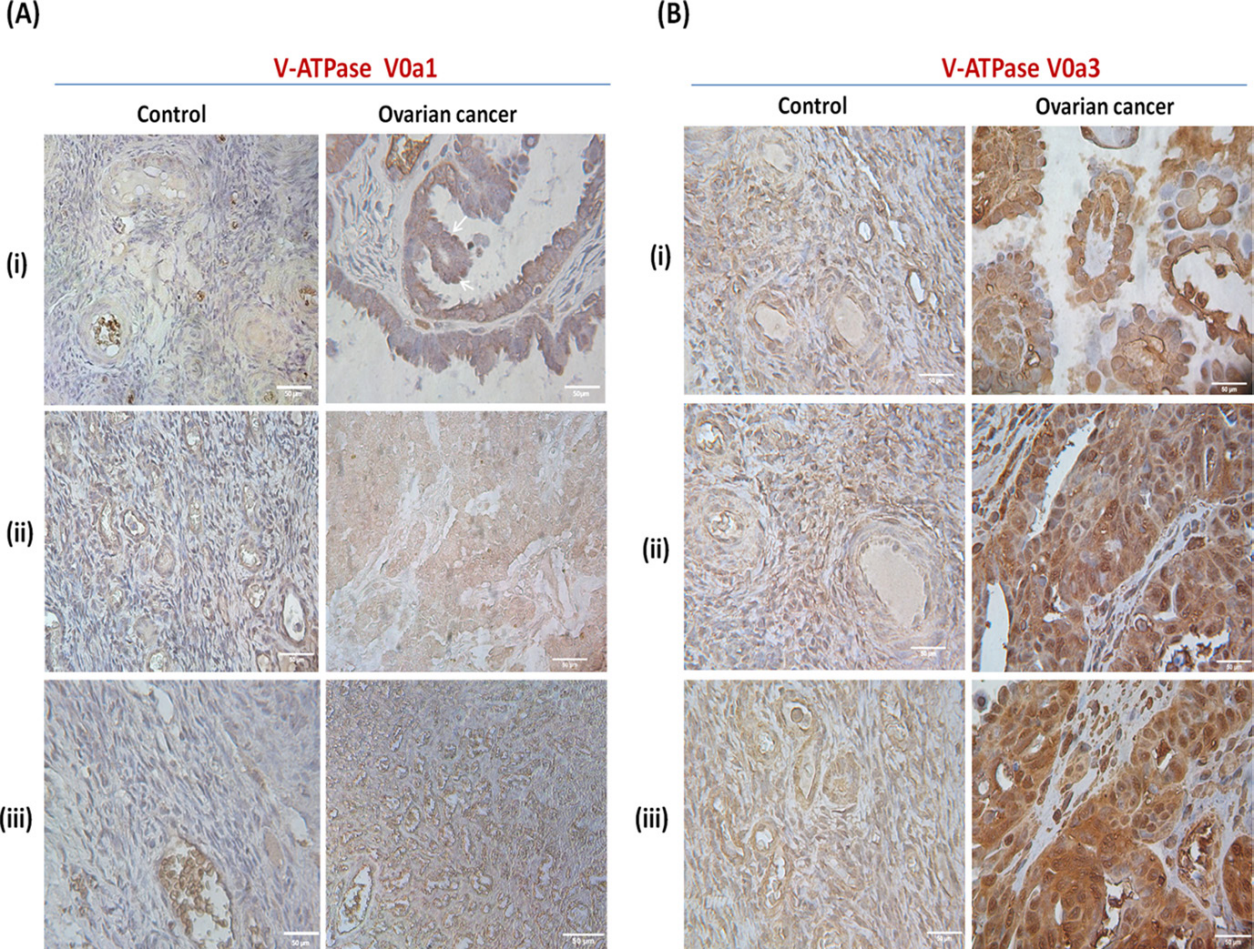
Expression pattern of V-ATPase V0a1 and V0a3 isoforms in ovarian cancer tissues Immunohistochemical analysis of V0a1 and a3 isoforms in human ovarian cancer tissues. **(A)** V0a1 expression was assessed using anti-V0a1 antibody against 74–93 amino acids. Control panel (i, ii)- Normal ovarian tissue, (iii)-benign granulosa of the ovary. Ovarian cancer panel (i) grade I serous cystedenocarcinoma. (ii) stage III endometroid cystedenocarcinoma, (iii) grade II serous cystedenocarcinoma. **(B)** V0a3 expresssion was assessed using anti-V0a3 antibody against 696–715 amino acids. Control panel shows (i), (ii)- Normal ovarian tissue, (iii)-benign granulosa cell tumor of the ovaries. Ovarian cancer panel shows (i) grade I serous cystedenocarcinoma. B(ii, iii) grade II serous cystedenocarcinoma. Unlike V-ATPase-V0a2 expression, the normal and benign OVCA tissues also exhibited some levels of V0a1 and V0a3 expression although, lower than metastatic tissues. Original magnification: × 400; scale bars –50 μm. The following Tissue Array was used: human ovary tumor tissue array; Biochain (Cat no: Z7020088).

To understand the clinical relevance of V0‘a1’ and a3 isoforms and their probable role in OVCA, immuno-histochemical analysis was performed. V-ATPase V0a1 was highly expressed in ovarian tumors compared to normal ovary epithelia [Figure 6(A)]. Interestingly, normal and benign tissues also stained positive for V0a1 expression, indicating a ubiquitous expression of V0a1 in normal ovarian cells and its role in normal cell physiology. V-ATPase V0a3 positive cells also expressed a higher level of expression in ovarian tumors when compared to normal tissues [Figure 6(B)]. However, benign tissues also expressed V0a3 in high amounts indicating that its expression does not solely indicate metastasis.

### V-ATPase- V0a2 inhibition decreases the activity of matrix metallo-proteases in ovarian cancer cells

**Figure 7:**
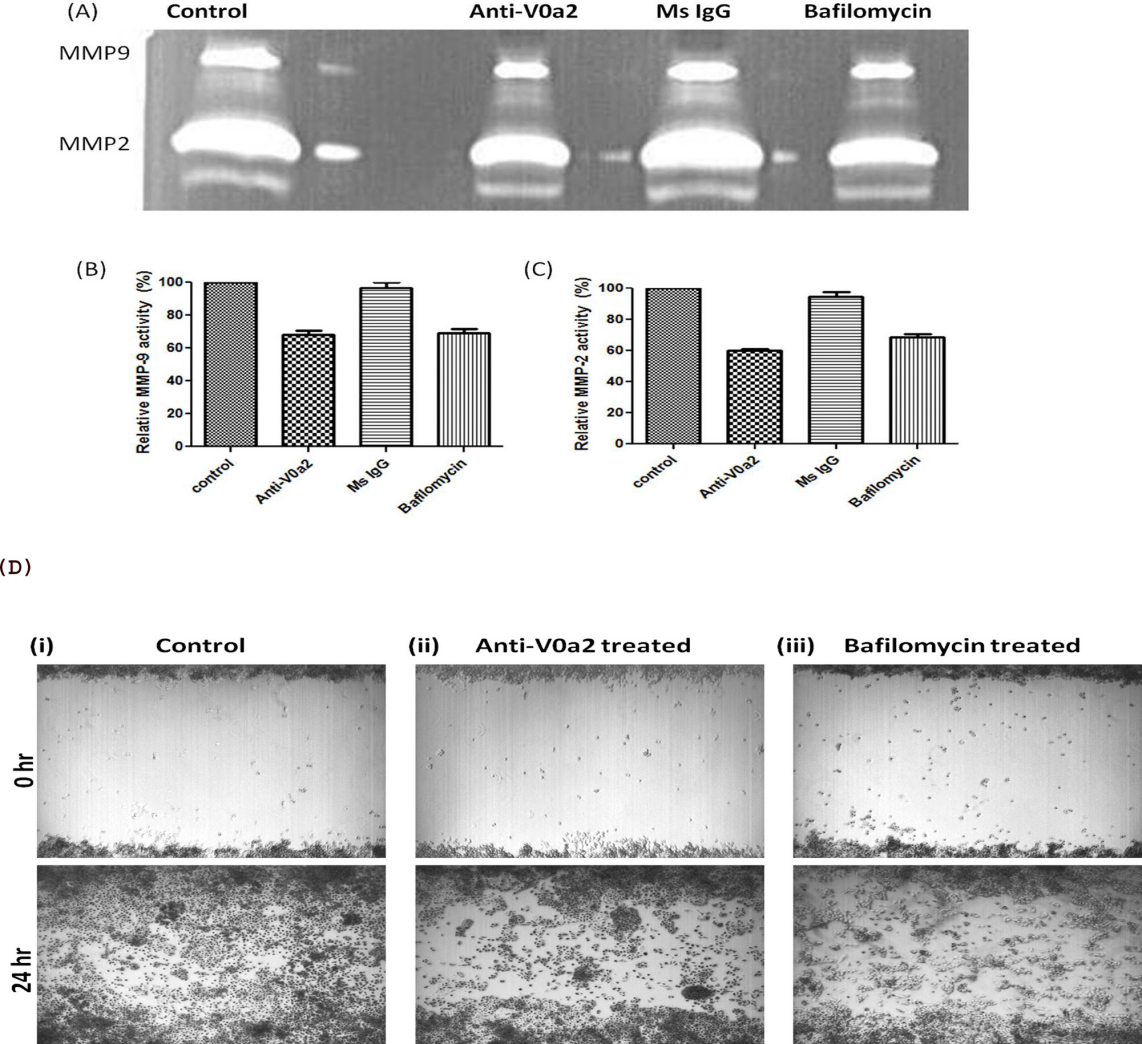
Inhibition of V-ATPase V0a2 decreases MMP-9 expression in ovarian carcinoma cells Ovarian cancer (OVCA) cell-derived MMP-9 and MMP-2 display reduced activities after v-ATPase-V0a2 isoform blockade. SKOV-3 OVCA cells were treated with Anti-V0a2 antibody (20ug/ml) along with untreated and Mouse IgG treated controls for 24 hr at 37°C in 5% CO2. For positive control, V-ATPase inhibitor, bafilomycin (200nM) was used for treating the ovarian cancer cells. Effects of v-ATPase inhibition on MMP-9 activity are shown. **(A)** Representative zymogram of anti-V0a2 treated SKOV-3 cell line is shown (top panel). There was a suppression in the **(B)** MMP-2 and **(C)** MMP-9 activity in anti-V0a2 antibody (20 μg/ml) treated SKOV-3 cells, comparable to bafilomycin. **(D)** Scratch/wound–healing motility assays over 24 h show that anti-V0a2 antibody treated SKOV-3 cells exhibit reduced migration at the wound edge. (i) control cells-Ms IgG (20 μg/ml) treated cells; (ii) Anti-V0a2 (20 μg/ml) treated cells; (iii) V-ATPase inhibitor, bafilomycin (200nM) treated cells. Original Magnification- X 40.

Matrix metalloproteinases (MMPs) are a family of zinc and calcium-dependent proteolytic enzymes that are critically involved in digesting the various components of ECM and are therefore necessary for tumor cell invasion. To determine the functional implications of V-ATPase and the V0a2 isoform inhibition on MMP activity, we performed zymography of cell culture supernatants from SKOV-3 OVCA cells treated with anti-V0a2 monoclonal antibody. Notably, we found that the activity of MMP-2 was much higher than the MMP-9 activity in ovarian cancer cells. The inhibition of V0a2 with anti-V0a2 (20 μg/ml) led to significant decrease in the activity of both MMP-2 and MMP-9 in the tested cell lines [Figure 7 (A–C)]. In order to validate the suppressed MMP activity functionally, tumor cell migration was investigated using a monolayer wound healing assay. Monitoring the cell movement over 24 h showed that migration was reduced in SKOV-3 cells by anti-V0a2 antibody treatment [Figure 7(D)]. These results indicate that ovarian cancer cells depend on the activity of V-ATPase V0a2 isoform for MMP activity.

## DISCUSSION

In tumor cells, due to intracellular acidosis, the increase in the activity of proton extrusion pumps such as vacuolar ATPases, is a survival mechanism to maintain the intracellular pH to normal levels [34–35]. The over-expressed V-ATPases in turn contribute to the acidified extracellular microenvironment which, ultimately promotes tumor metastasis [36–37] and drug resistance [38]. In many studies, over-expressed V-ATPases are related to a highly invasive tumor phenotype [17, 23, 39]. New strategies are therefore being explored for targeted impairment of this multi-subunit enzyme in cancer [3, 40–41]. Nevertheless, it is pivotal to understand the role of this multi-tasking enzyme in ovarian cancer (OVCA), for which, effective strategies are acutely needed to improve the associated mortality rates. The current study highlights the involvement of the ‘V0a2’ isoform of V-ATPase on the surface of metastatic ovarian tumors and its co-association with cortactin, a component of invasion machinery at the advancing edge of the tumor cell surface. Targeted impairment of V-ATPase-V0a2 reduces the activity of matrix metallo-proteinases, such as MMP-2 and MMP-9, in OVCA cells. This further supports its association with plasma membrane V-ATPase that mediate extracellular acidification. These observations suggest that it can be a useful target for developing biomarkers and for targeted therapeutics against OVCA. To the best of our knowledge, this is the first report on the expression of V-ATPase ‘a’ subunit in ovarian carcinoma.

The localization of V-ATPase is crucial as it determines its role in vesicular trafficking, molecular transport and protease activation. Here, we report an elevated expression of V-ATPase-V0a2 on the surface of malignant cells in a panel of human ovarian carcinoma tumor tissues compared to normal ovaries or benign tumors, suggesting the potential of V-ATPase-V0a2 as a diagnostic marker for metastatic ovarian tumors. This data supports our previous observation which, postulated that V0a2 moves to the cell surface in response to acidic cell stress [42]. Confocal microscopy and flow cytometry examination further demonstrated that in ovarian carcinoma cell lines, V-ATPase ‘V0a2’ is highly expressed on the plasma membrane indicating that this isoform is one of the components of membrane bound V-ATPase involved in acidifying extracellular microenvironment of tumor cells. Interestingly, the normal cells did not express V0a2 on cell surface indicating that the membrane bound V0a2 has a specific role in tumor metastasis.

Plasmalemmal V-ATPases are known to play a role in tumor metastasis [43]. Our previous studies have indicated that V-ATPase, specifically, the V0a2 isoform is important in promoting tumor invasion and angiogenesis [24–25]. Several roles of V-ATPases-V0a2 in tumor growth have been suggested. Firstly, it controls the hydrogen ion production on the tumor cell surface for maintenance of pH and acts as a surface ATPase [44]. Secondly, our recent studies have shown that V0a2 located on tumor cell surface is cleaved at N-terminal domain which is then secreted into extracellular environment. This creates a pro-inflammatory environment by stimulating tumor associated macrophages which further secrete high levels of IL-1 beta, VEGF, MMPs production promoting angiogenesis and growth in tumors [24–25, 30, 44]. Finally, it is also postulated that under acidic conditions, V-ATPase-V0a2 is sorted to the tumor cell surface with simultaneous up-regulation of cytokine secretion and microvesicle generation [42, 44, 45].

Tumor metastasis is attributed to an increased migratory capacity of the cells [32]. The leading edges/protrusions are formed on tumor cell surface by localized polymerization of F-actin filaments that assist invasion. These leading edges are also the focal points for release of matrix degradative proteases associated with tumor cell invasion. Cortactin is a tyrosine kinase substrate that plays a central role stabilizing the F-actin filaments at the leading edges of tumor cells and controls protease release at invasion sites [33, 46]. We observed a co-association of V-ATPadse-V0a2 with cortactin filaments in specific regions of the cell surface. This indicates that V0a2 is a part of the membrane bound V-ATPase machinery related to the tumor cell invasion and is also a potential target for anti-invasive therapy.

Further, we found an association of V0a2 isoform with the endosomal machinery. V-ATPases activity influences endosomal acidification, that further regulates other important processes such as membrane trafficking, receptor-ligand dissociation, recruitment of signal molecules, intracellular V-ATPase distribution as well as the activation of lysosomal enzymes [8–9; 47–48]. This has an overall impact on tumor growth and metastasis via the modulated signals and their pathways. Crucial tumor associated pathways such as Notch, wnt, mTOR [49–50] are modulated by V-ATPase through endosomal trafficking and pH maintenance. The colocalization of V0a2 isoforms with endosomes suggests that it may be a component of endosomal V-ATPase machinery and can be targeted for modulating the tumor associated signaling pathways.

In line with a recent report [51] we observed that V0a1 isoform has a surface expression in OVCA cells. However, this isoform was also expressed on normal ovary cell surfaces, suggesting that its presence on cell surface is ubiquitous and is associated with normal cell physiology. Interestingly, in contrast to previous reports [23] where the expression of V0a3 was specifically confined to surface of highly invasive melanoma cells, the present data indicates that V0a3 expression is mainly intracellular in the studied OVCA cell lines. Taken together, among all V-ATPase-V0‘a’ isoforms, V0a2 is a potential candidate for the targeted inhibition of membrane bound V-ATPases on cancer cells.

One of the mechanisms to promote tumor invasion involves modulation of extracellular protease activity. Matrix metalloproteinases (MMPs) secreted by cervical and ovarian cancer, especially MMP-2 and MMP-9, play crucial roles in tumor invasion and metastasis. In invasive pancreatic cancer cell lines, MMP-9 activity was reduced with V-ATPase blockade where V-ATPase was localized on plasma membrane, but was least affected in cells which demonstrated little V-ATPase plasma membrane localization [35]. Cortactin containing leading edges on tumor cell surface have been implicated in focal MMP release and our data indicates that V-ATPase-V0a2 is a potential part of this invasion machinery. Furthermore, the inhibition of V-ATPase-V0a2 using monoclonal anti-V0a2 antibody (2c1) led to reduced MMP-2 and MMP-9 activity. Inhibitory activity of anti-V0a2 antibody (also known as anti-RTF antibody) is well established in both *in vitro* and *in vivo* systems in our previous reports where this antibody has been shown to inhibit the V-ATPase function in macrophages [43] as well as in activated T cells where neutralizing anti-RTF antibodies induced apoptosis in activated T cells [52]. Further, in murine male reproductive system, inhibition of V0a2 using anti-a2v antibody injection in males led to poor pregnancy outcome in females [53]. The advantage of isoform specific antibody for targeted inhibition of V-ATpase is that unlike other chemical reagents such as macrolide antibiotics (bafilomycinA, concanamycinA etc.) that also block the function of intracellular V-ATPases, this antibody specifically recognizes the functional membrane bound form of V-ATPases present on malignant cells. This will help reduce the associated cytotoxicity to the normal cell population.

In conclusion, we have shown that V-ATPase-V0a2 isoform is abundantly expressed on ovarian tumor cell surface in association with proteins involved in invasion related assembly on plasma membrane and plays a critical role in tumor invasion and metastasis by modulating the activity matrix degrading proteases. The knowledge from the present study gives us the opportunity to develop new anticancer drugs aimed against V-ATPase-V0a2 as a specific target exhibiting distinct expression in ovarian cancer.

## MATERIALS AND METHODS

### Cell lines and cell culture

Three human ovarian adenocarcinoma cell lines A2780, TOV112D and SKOV-3 were employed in the study. A2780 cell line (Sigma Aldrich, USA) was cultured in RPMI (Invitrogen, Carlsbad, CA), TOV112D cell line (American Type Culture Collection [ATCC], Manassas, VA) was cultured in CTOV medium [1:1 mixture of MCDB 105 medium containing a final concentration of 1.5 g/L sodium bicarbonate and Medium 199 containing a final concentration of 2.2 g/L sodium bicarbonate], SKOV-3 cells (ATCC) were cultured in Eagle MCoy 5 medium (Hyclone, UT, USA). All media were supplemented with 10% FBS, 1% L-glutamine, and 1% penicillin and streptomycin. Cells were cultivated at 37°C in a humidified atmosphere containing 5% (v/v) CO_2_. For routine culture, cells were grown until reaching approximately 80% confluency and then subcultured or plated for experiments.

### RNA isolation and reverse transcription-PCR

For RNA extraction, cultured cells were washed with HBSS (Gibco, USA) and harvested using trypsin-EDTA (Sigma, USA). RNA isolation was performed using RNeasy^®^ mini kit (Qiagen, CA, USA) according to the manufacturer's protocol. Samples were stored at −80°C until further use. 2.5 micrograms of total RNA was reverse transcribed at 37ºC using random primers and M-MLV Reverse transcriptase system using high capacity cDNA kit (Applied Biosystems, Foster City, CA, USA) using conditions recommended by the manufacturer. At least three biological replicates were prepared for each of the samples. Duplex RT-PCR was performed using the Step One Real-Time PCR system (Applied Biosystems), with GAPDH as the internal reference. The prevalidated TaqMan gene-expression assays for *V0a1* (*Atp6v0a1*; Hs00193110_m1); *V0a2* (*Atp6v0a2*; Hs00429389_m1); V0a3 (Hs00990751_m1) *V0a4* (Hs00220886_m1), and internal control *Gapdh* (4326317E) were purchased from Applied Biosystems (Foster City, CA). All Real time PCR reactions were performed in triplicate in 10 μl volumes using Universal fast PCR Master Mix reagent (Applied Biosystems) according to the manufacturer's protocol.

### Antibodies

The human anti-V0a2 antibody against 488–510 amino acids of trans-membrane region (2C1) was employed as described previously [24–25]. For V0a1 and V0a3, antibodies were raised in rabbit against the synthetic peptides from unique regions of human V0a1 (amino acids 73–95; RKANIPIMDTGENPEVPFPRD) and V0a3 (amino acids 696–715; EEKAGGLDDEEEAELVPSEVL) by Covance (USA) and V0a1 antibody was affinity purified using Protein-G by Covance. The V0a3 antibody was affinity purified using a melon-Gel IgG purification kit (Thermo Scientific, Rockford, USA).

### Immunohistochemical staining of ovarian cancer tissue

Paraffin embedded ovarian cancer and normal ovarian tissue sections were obtained from Biochain Institute, Inc (Newark, CA, USA) and were stored at 4°C until used. The arrays were stained using a method based on horseradish peroxidase-labeled polymer (EnVision+ Dual Link System-HRP; DAKO, USA) according to manufacturer's protocol, preceded by an antigen retrieval procedure by boiling the sections in sodium citrate buffer (pH = 6.0) as described previously [28]. Experiments were performed at least in duplicate.

For detection of V0a1, V0a2, V0a3 protein, sections were incubated with 15 μg/ml, 5 μg/ml and 28 μg/ml of IgG antibodies respectively, in 1% BSA-PBS overnight at 4°C. Simultaneously, for controls, tissue sections were stained with mouse/rabbit isotype-control antibodies (R&D systems, USA) using at the same concentration as the primary antibodies. The sections were counterstained with Mayer's hematoxylin and mounted in Faramount aqueous mounting medium (Dako). The immunostaining was evaluated by light photomicroscopy (Carl Zeiss, Weesp, The Netherlands) using a high-resolution camera (Canon G10, Canon, Tokyo, Japan). Scale bars were calculated using ImageJ software.

### Immuno fluorescence analysis

Ovarian carcinoma cell lines were plated in 8-well chamber slides (Nunc, USA) at 0.25 × 10^4^ cells/well and were incubated for 24 hrs in 5% CO2 at 37ºC. Approximately 24 h later, cells were washed thrice with PBS, fixed with 4% paraformaldehyde for 15min, and permeabilized with 0.1% Triton X-100 in PBS fo 10min at RT. Nonspecific binding was blocked by incubation with 3% fetal bovine serum in PBS for 1 h at RT and then incubated for 1hr at RT with the anti- a1/a2/a3 subunit antibody along with the antibodies targeting the organellar markers anti-pan cadherin (1:100) for plasma membrane, anti-Rab5 (1:250) or anti-EEA-1 (1:250) for early endosome anti-cortactin (1:25), anti-calnexin (1:300) for endoplasmic reticulum, anti-Golph-4 (1:100) for Golgi (Abcam, USA). Anti-rab7(1:200) antibody was obtained from Cell Signalling technology, USA. The cells were next rinsed three times with PBST and then incubated with Alexa Fluor^®^ 488-conjugated goat anti-rabbit or Alexa Fluor^®^ 594-conjugated goat anti-rabbit secondary antibody (1:200 dilution) (Invitrogen) in 3%FBS in PBS. After 45 min of incubation at room temperature, the cells were again rinsed with PBST. The cells were prepared for viewing using ProLong^®^ Gold (Invitrogen) mounting medium containing DAPI and allowed to polymerize at room temperature for 24 h. For confocal microscopy, the stained cells were imaged on a Olympus Fluoview Fv10i confocal microscope. Analysis was performed using Fv10i Flouview Ver.3.0 software. Experiments were repeated at least twice in duplicate. For immunofluorescence microscopy, stained cells were imaged in Olympus microscope and analyzed using NIS-Elements Ar software (Nikon Inc, NY USA).

### Flow cytometry analysis

Ovarian caricinoma cells and normal ovarian surface epithelial cells (2.5 × 10^5^ cells/tube) were washed with HBSS containing 0.1% FBS. For surface staining of the VATPase V0a2 subunit isoform, the cells were incubated with mouse monoclonal V0a2 Ab conjugated to Alexa Fluor 647 (Covance, Denver, PA) in HBSS containing 0.5% FBS for 40 min at 37°C. Similarly, for intracellular staining, the cells were fixed and permeabilized using fixation and permeabilization buffer (BD Biosciences, San Jose, CA, USA) and the cells were stained as described above. For V0a1 and V0a3 subunit isoforms, indirect staining was performed. The cells incubated with antiV0a1 or antiV0a3 rabbit polyclonal IgG1antibody for 1 hr at 37°C and after washing 2 times with HBSS/0.1% FBS, the cells were stained with donkey Alexa Fluor 647 anti-rabbit IgG secondary antibody (Abcam, USA) for 30 min at 37°C. Appropriate isotypes (Rabbit or mouse primary antibody isotype controls) and unstained controls were used for each set of experiments. The stained cells were analyzed on a BD LSR II flow cytometer with FlowJo software (Tree Star). Experiments were performed twice in duplicate.

### Western/Dot blot analysis

Cells were harvested, resuspended in lysis buffer with protease inhibitors (Pierce Protein Biology, USA) and lysed by protein lysis bufer. Cell lysates were centrifuged for 30 min at 4°C at 13,000 × rpm to remove cellular debris. Protein concentrations were determined using Bradford assay. SDS sample buffer was added to the lysates and aliquots containing 40 μg of protein were separated by SDS-PAGE on 4–20% gradient acrylamide gels. For detecting the presence of subunit ‘a’ isoforms or endogenous control beta-actin, antibodies against V0a1/V0a2/V0a3 as described previously were used followed by IR secondary antibody (Licor) and visualized using Odyssey^®^ infrared imaging system (LI-COR Biotechnology). For dot blot analysis, synthetic peptides of each of the V0‘a’ isoforms (V0a1, Va2, V0a3) from which the antibodies were generated, were used. The peptides were spotted on the nitro-cellulose membrane (Sigma, USA). The membrane was allowed to air-dry and subjected to immuno-blotting and analysis as described above.

### Gelatin zymography

The conditioned media from anti-V02 antibody treated OVCA cells was mixed with sample buffer (2X) and applied directly, without prior heating or reduction, to 10% zymogram gels containing 1 mg/ml gelatin. After electrophoresis, the SDS was removed from the gel by incubating in 2.5% (v/v) Triton X-100 (Sigma) for 30 min. The gels were then incubated at 37°C overnight in development buffer (50 mmol/l Tris-HCl, pH 7.6, containing 0.2 M NaCl, 5 mmol/l CaCl2) for 24 h at 37°C. Gels were then stained with 0.2% Coomassie blue. White bands representing gelatinase activity were photographed in gel documentaion system (Biorad, CA, USA).

### *In vitro* wound healing assay

For scratch assay, SKOV-3 cells were grown on 6 well plates till they formed a monolayer. A line was drawn with a marker on the bottom of the well. Once the cells formed monolayer, three separate wounds were scratched through the cells moving perpendicular to the line drawn using a sterile 1000 μl pipet tip. The cells were gently washed and fresh media was added. The images were captured at the beginning (0 hr) and 24 hr during cell migration to close the scratch, and compared to analyze the migration of the cells.

### Statistical analysis

All experiments were conducted at least in triplicate and results are expressed as mean ± SD. Student's unpaired t test was used to determine the statistical significance of the values obtained using Graph pad prism 5.0 software. Statistical significance was accepted as *p* < 0.05. For immunohistochemistry images, scale bars were calculated using ImageJ software.

## SUPPLEMENTARY FIGURES


