# Supplementary material for: Vacuolar ATPase ‘a2’ isoform exhibits distinct cell surface accumulation and modulates matrix metalloproteinase activity in ovarian cancer
Source: Oncotarget. 2015 Jan 21;6(6):3797–810. doi: 10.18632/oncotarget.2902 (PMC4414154; doi:10.18632/oncotarget.2902)
Supplement: Supplementary file 1 [file oncotarget-06-3797-s001.pdf]

SUPPLEMENTARY FIGURES

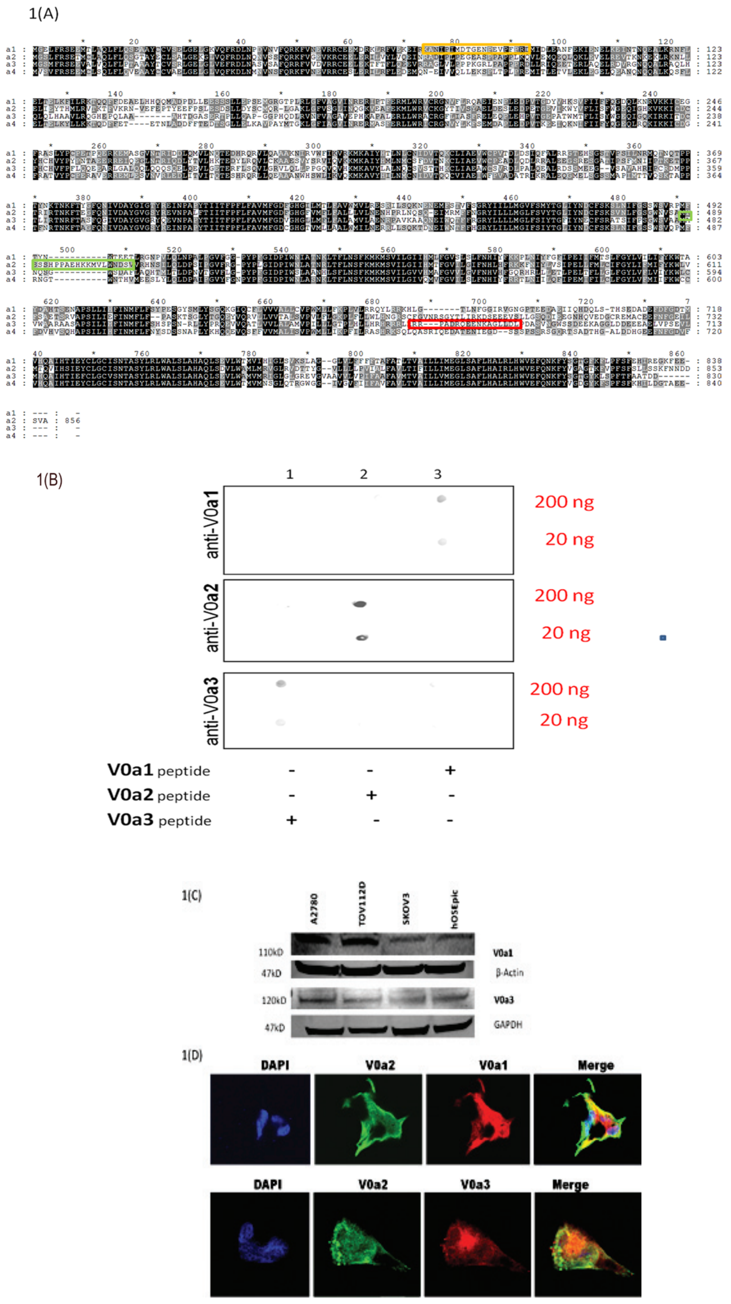

Supplementary Figure S1: Sequences of V-ATPase V0'a' subunit isoform specific epitopes selected for antibody production. (A) Anti-V0a1 antibody was designed against 74–93 amino acids of the human V-ATPase V0a1 sequence (marked in yellow). Anti-V0a2 antibody was designed against 488–510 amino acids of trans-membrane region of the human V-ATPase V0a2 sequence (marked in green). Anti-V0a3 antibody was designed against human V-ATPase 696–715 amino acids of the V0a3 sequence (marked in red). All these selected regions are isoform specific as determined by BLAST analysis. **(B)** A dot blot analysis was performed for the human V0'a' isoform antibodies using specific peptides from which these antibodies were generated. V-ATPase V0a3 peptide (lane 1), V-ATPase V0a2 peptide (lane 2) and V-ATPase V0a1 peptide (lane 3) using peptide concentrations 200ng and 20ng. The dot blot indicates that each of the V-ATPase V0'a' isoform specific antibodies is specific for their corresponding peptides and do not cross react with one another at the tested concentrations. **(C)** western blot analysis showing V-ATPase V0a1 and V0a3 proteins with band size near 116KD in Ovarian carcinoma cell lines. **(D)** Sub-cellular distribution in ovarian cancer cells by confocal immunofluorescence microscopy using the V0'a' isoform specific antibodies reveals specific expression pattern of the V0a isoforms using these antibodies and these do not cross react. Nuclear staining shown using DAPI (blue). Representative TOV-112D cells used here. Maginification- X600.

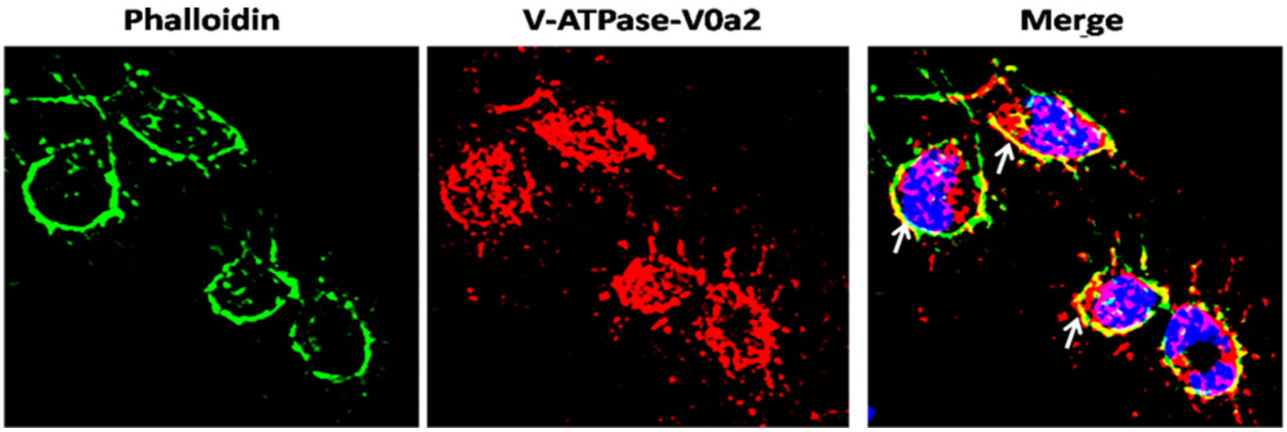

Supplementary Figure S2: V-ATPase V0a2 isoform is co-associated with F-Actin cytoskeletal machinery in ovarian cancer. F-actin labeled with fluorescent conjugated phalloidin (green). Anti-V0a2 stain V-ATPase-V0a2 isoform (red); Merged image, demonstrates co-distribution (yellow) in certain areas.

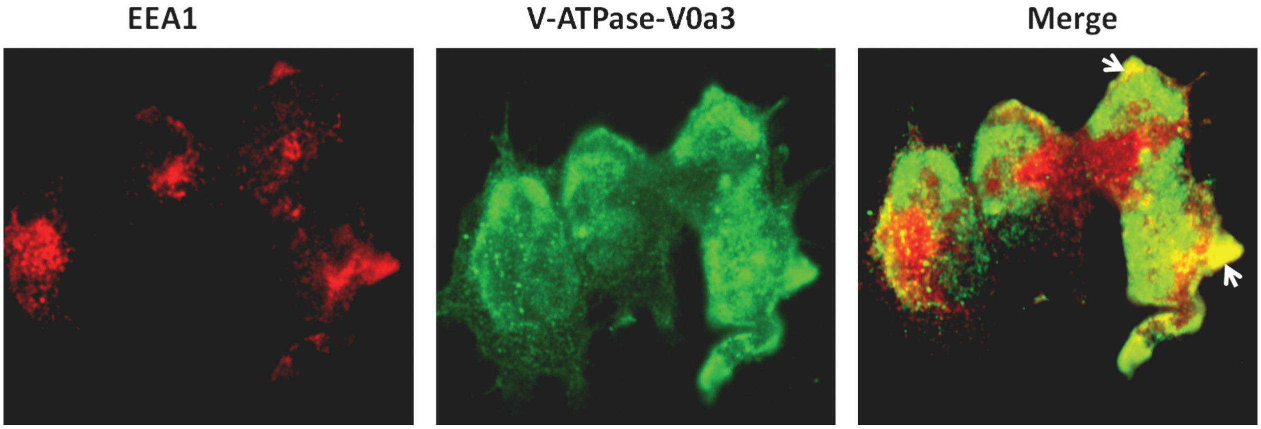

Supplementary Figure S3: Expression and localization of V-ATPase V0a3 subunit in ovarian cancer. Anti-EEA1 antibody stains the early endosome (red), anti-V0a3 was used to stain V0a3 (green); Merged image, demonstrates co-distribution (yellow).
